# Blowfly-derived mammal DNA as mammal diversity assessment tool: Determination of dispersal activity and flight range of tropical blowflies

**DOI:** 10.3897/BDJ.11.e108438

**Published:** 2023-09-12

**Authors:** Ping Shin Lee, Min Hui Dong, Xin Lei Yan, Tian Yi He, Shang Fei Yu, Suk Ling Wee, John James Wilson

**Affiliations:** 1 College of Life Sciences, Anhui Normal University, Wuhu 241000, Anhui, China College of Life Sciences, Anhui Normal University Wuhu 241000, Anhui China; 2 Anhui Provincial Key Laboratory of the Conservation and Exploitation of Biological Resources, Anhui Normal University, Wuhu 241000, Anhui, China Anhui Provincial Key Laboratory of the Conservation and Exploitation of Biological Resources, Anhui Normal University Wuhu 241000, Anhui China; 3 Centre for Insect Systematics, Faculty of Science and Technology, Universiti Kebangsaan Malaysia, 43600 Bangi, Selangor, Malaysia Centre for Insect Systematics, Faculty of Science and Technology, Universiti Kebangsaan Malaysia 43600 Bangi, Selangor Malaysia; 4 Department of Biological Sciences and Biotechnology, Faculty of Science and Technology, Universiti Kebangsaan Malaysia, 43600 Bangi, Selangor, Malaysia Department of Biological Sciences and Biotechnology, Faculty of Science and Technology, Universiti Kebangsaan Malaysia 43600 Bangi, Selangor Malaysia; 5 Vertebrate Zoology at World Museum, National Museums Liverpool, William Brown Street, Liverpool, United Kingdom Vertebrate Zoology at World Museum, National Museums Liverpool, William Brown Street Liverpool United Kingdom

**Keywords:** dispersal range, blowflies, iDNA, mammal diversity, tropics

## Abstract

Mammalian DNA extracted from the invertebrates, especially blowfly-derived DNA, has been suggested as a useful tool to complement traditional field methods for terrestrial mammal monitoring. However, the accuracy of the estimated location of the target mammal detected from blowfly-derived DNA is largely dependent on the knowledge of blowflies' dispersal range. Presently, published data on adult blowfly dispersal capabilities remain scarce and mostly limited to temperate and subtropical regions, with no published report on the adult blowfly dispersal range in the Tropics. We seek to determine the blowfly flight range and dispersal activity in a tropical plantation in Malaysia by mark-release-recapture of approximately 3000 wild blowflies by use of rotten fish-baited traps for nine consecutive days. Out of the 3000 marked *Chrysomya* spp., only 1.5% (43) were recaptured during the 9-day sampling period. The majority of the blowflies (79%) were recaptured 1 km from the release point, while 20.9% were caught about 2-3 km from the release point. One individual blowfly travelled as far as 3 km and before being recaptured, which was the maximum dispersal distance recorded in this study. This result suggests that the estimated locations of the mammals detected from blowfly-derived iDNA is likely to be within 1-2 km radius from the origin of the blowfly sampling location. However, a more accurate estimated distance between the target mammal and the blowfly sampling location requires further investigation due to various factors, such as blowfly species, wind speed and direction that may potentially affect the blowfly dispersal activities. This study contributes further understanding on the development of a blowfly-derived DNA method as a mammalian monitoring tool in the tropical forests.

## Introduction

Invertebrate-derived DNA (iDNA) has recently been suggested as an alternative to traditional field methods for surveying and monitoring mammalian biodiversity (Schnell et al. 2012, Calvignac‐Spencer et al. 2013, Calvignac-Spencer et al. 2013, Lee et al. 2015, Schnell et al. 2015, Rodgers et al. 2017, Hoffmann et al. 2018, Abrams et al. 2019). Invertebrates that come into contact with vertebrates or their by‐products as part of their daily activities represent a promising source of vertebrate DNA for reliable metabarcoding‐based assessments of terrestrial biodiversity (Gogarten et al. 2019, Srivathsan et al. 2022). Blowflies may have advantages over other sources of iDNA for terrestrial mammal monitoring (Calvignac-Spencer et al. 2013), such as leeches that are habitat-restricted (Schnell et al. 2012, Schnell et al. 2018, Siddall et al. 2019), ticks that feed infrequently (Humair et al. 2007, Gariepy et al. 2012) and mosquitoes and tsetse flies that have narrow host preferences (Kent and Norris 2005, Lyimo and Ferguson 2009, Muturi et al. 2011, Kocher et al. 2017, Reeves et al. 2018), due to their unique behaviour, biology and ecology. For example, an iDNA study using blowflies detected small- to large-bodied mammals, including volant and non-volant species in tropical forests of Malaysia (Lee et al. 2016). In addition to high dispersal capability and broad feeding preferences (Bishopp and Laake 1921, Azwandi et al. 2013, Calvignac-Spencer et al. 2013, Lee et al. 2015, Rodgers et al. 2017), blowflies usually arrive in large numbers at animal carcasses and faeces in almost all habitats, which not only pick up host DNA effectively (Norris 1965, Owings et al. 2019), but also increase the chances of being detected.

The mobility of the iDNA-carrying fly species could impact the spatio-temporal resolution of the iDNA data (Srivathsan et al. 2022). Considering the temporal persistence of mammal DNA in blowfly guts (Lee et al. 2015) and that the blowfly-derived DNA approach has been field-calibrated against other traditional field methods (Lee et al. 2016), appropriate methods for blowfly sampling have been recently suggested (Calvignac‐Spencer et al. 2013, Calvignac-Spencer et al. 2013, Lee et al. 2016). However, there has been no unified standard on how fly traps should be set up in the field for mammal monitoring studies. For example, traps by Rodgers et al. (2017) were set up along transects at an interval of 250 m, traps by Gogarten et al. (2019) were set up in one-km intervals along the grid system and fly traps were set up densely at varying distances from a road in the forest (Srivathsan et al. 2022). In addition, some uncertainties, such as blowfly dispersal relative to the location of species detected from blowfly-derived DNA, remain to be addressed (Calvignac-Spencer et al. 2013, Schnell et al. 2015, Lee et al. 2016).

Knowledge of the invertebrate dispersal range is essential for estimating the location of the mammal species relative to the location where the invertebrates were collected (Schnell et al. 2015, Srivathsan et al. 2022). Inadequate information on flight range and dispersal activities of blowflies, in particular, can result in great uncertainties regarding the precise location of mammal species detected from blowfly-derived DNA (Schnell et al. 2015). Blowflies are thought to disperse long distances due to their strong flight ability (Bishopp and Laake 1921), relative to other invertebrates, such as leeches that exhibit little movements (Calvignac-Spencer et al. 2013, Schnell et al. 2015). However, data on adult blowfly dispersal capabilities are surprisingly scarce (Braack and Retief 1986, Amat et al. 2016). Studies suggested that the daily dispersal capabilities of blowflies from the temperate and subtropical regions are 0.10-0.15 km and 1.25-2.35 km, respectively (Braack and Retief 1986, Smith and Wall 1998, Tsuda et al. 2009; Table 1). However, there were no published data on blowfly dispersal ranges in the Tropics. Previous studies of the dispersal of adult dipterans (Calliphoridae, Sarcophagidae, Muscidae, Drosophilidae and Tephritidae) by marking techniques, study locations and dispersal ranges are summarised in Table 1. From these data, there is a clear difference in the dispersal range of blowflies in terms of species and regions, with environmental conditions acting as barriers to some species (MacLeod and Donnelly 1960, Tsuda et al. 2009).

Considering the implications of the dispersal capabilities of blowflies (Family: Calliphoridae) might have on the development of a mammal monitoring tool via blowfly-derived DNA, we seek to determine the dispersal activities and flight range of blowflies in the tropical forests by conducting a mark-release-recapture study of *Chrysomya* spp. in a selected plantation in Malaysia.

## Material and methods

**Study site**

**Figure 1.**
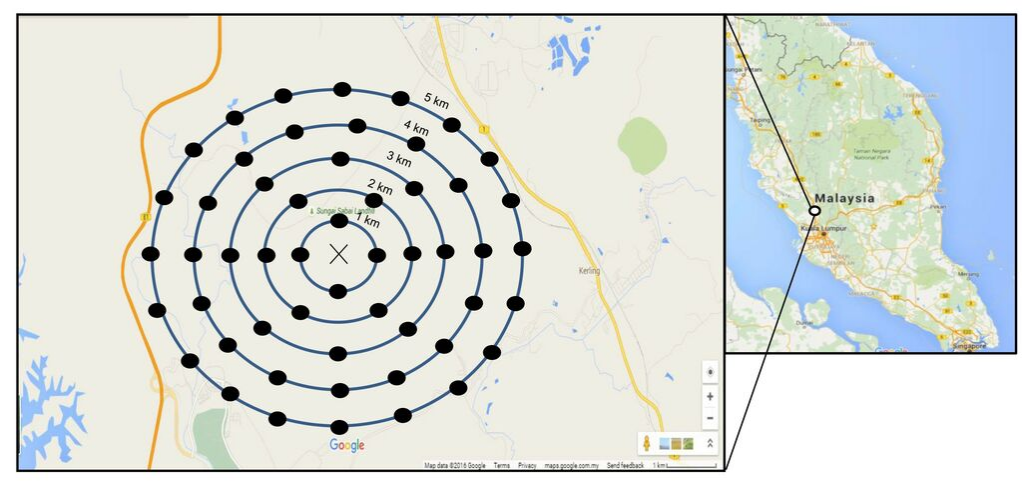
The map is showing the location of the rubber plantation, Kalumpang, Selangor where the fieldwork of dispersal range of blowflies is conducted. Inset showing the mark-release-recapture experimental design, with X denoting the release point of blowflies and solid dots represented recapture points by using rotten fish-baited traps.

Our study was conducted at a rubber plantation in Kuala Kalumpang, Selangor (Fig. 1). Kuala Kalumpang (3°36'N 101°33'E) is located about 4.8 km south of Tanjung Malim, with two small towns, Kalumpang and Kerling connected by highways. It comprises tropical rainforest, especially in the Titiwangsa Range of Peninsular Malaysia including Bukit Kalumpang. Some of the areas are covered with rubber plantations, oil palm plantations and orchards (Omar 1981). The rubber plantation is suitable for insect dispersal studies as it provides a large scale of surface area with ease of access for sampling (Franzén and Nilsson 2007, Hassall and Thompson 2011) and has an equatorial climate which is classified as rainforest climate according to the Köppen classification (Kottek et al. 2006). The annual temperature range in Kalumpang is 21-33°C (Meteoblue 2023) with a high humidity (80%-90%) and annual rainfall of 2,850 mm and two distinct wet seasons occur in April-May and September-November (Nieuwolt 1982).

**Collection and marking of blowflies**

Adult blowflies were collected using traps baited with ca. 200 g rotten fish (hereafter referred to as blowfly traps) (Lee et al. 2015) within the campus of University of Malaya, Kuala Lumpur and Kampong Ulu Dong, Pahang between 17 December 2015 and 26 December 2015. Flies were brought back to the laboratory for morphological identification under a stereomicroscope up to *Chrysomya* genus (Kurahashi et al. 1997). The identified wild *Chrysomya* blowflies were carefully transferred into ten cages (39 x 25 x 33 cm; approximate 300 blowflies each cage) by using specimen vials. The blowflies were then provided with sugary solution and maintained at room temperature (27°C-33°C) and relative humidity 70-80%. One day prior to release to the field, the blowflies were marked by orange-coloured fluorescent dust (Transcend Solutions-Selangor, Malaysia) by mass dusting (Howard et al. 1989, Nazni et al. 2005). This method has been commonly used in most of the conventional mark-release-recapture studies of insects (Hagler and Jackson 2001). The fluorescent dust remains detectable for the duration of the life of flies or at least 28 days under natural conditions (Pickens et al. 1967, Moth and Barker 1975, Lillie et al. 1981). Most importantly, the technique does not affect the survival of the flies (Pickens et al. 1967, Moth and Barker 1975, Chiang et al. 1991).

**Release and recapture of blowflies**

On 27 December 2015, the marked blowflies, approximately 3,000 individuals, were released at 10:00 h, i.e. within the active flight activity of blowflies (Das et al. 1978, George et al. 2012), in the selected rubber plantation at Kalumpang, Selangor (Fig. 1). Recapture of blowflies commenced 24 h after release and continued for nine consecutive days (following Howard et al. (1989), Chiang et al. (1991), Smith and Wall (1998)). The weather conditions throughout the sampling period were mostly cloudy with slight or no rain. Daily temperatures during the study period were between 20.7°C and 34.5°C, with dominant northeast wind (Malaysian Meteorological Department 2023). Blowfly traps were set at 2 m above ground in five concentric radii of 1, 2, 3, 4 and 5 km with the release point at the centre. A total of 57 traps were set up, with the number of traps per circle increased with the increase of every 1 km distance from the release point (Fig. 1). Captured blowflies were collected from the traps daily between 10:30 and 12:30 h and stored at 0°C for further examination.

**Identification of trapped flies**

Captured blowflies were examined for the presence of fluorescent powder on their bodies under ultraviolet (UV) light in a dark room. The number of marked blowflies recaptured at different days-after-release (DAR) and distance from the release point were recorded accordingly (Suppl. material 1).

## Results

**Figure 2.**
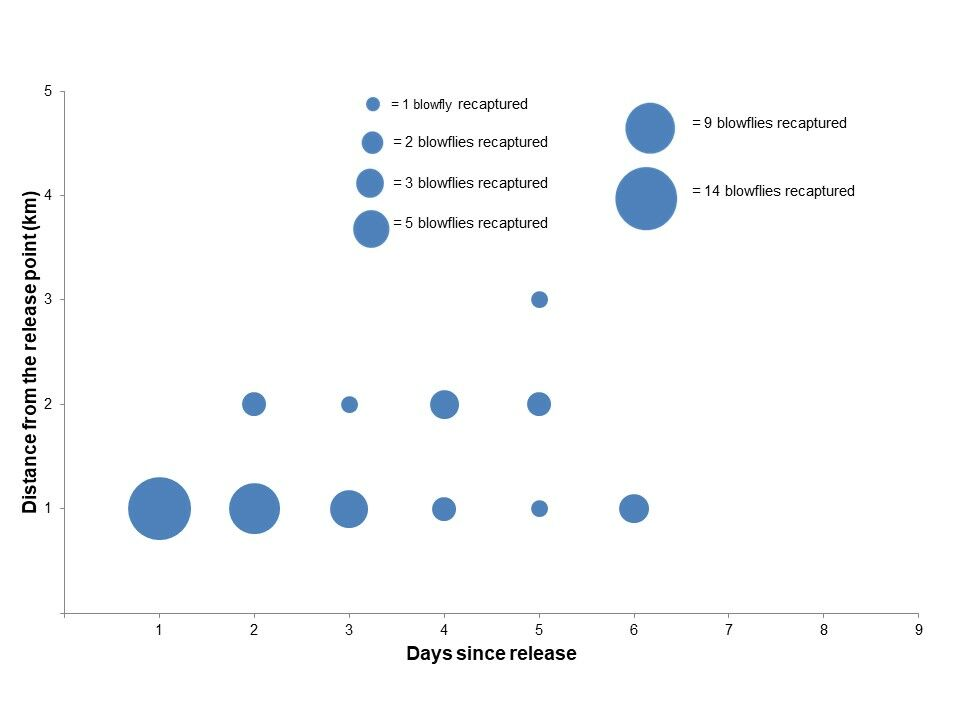
The number of blowflies recaptured at different days-after-release (DAR) and distance from the release point (1-5 km). The size of the circle indicates the number of blowflies recaptured.

Forty-three *Chrysomya* spp., representing 1.5% of the total released, were recaptured between 1 3 km radius from the release point during the 9-day experimental period (Fig. 2). Of these, 34 individuals (79%) and eight individuals (18.6%) were recaptured at 1 and 2 km radius from the release point, respectively. Only one individual (2.3%) was recaptured at 3 km distance (Fig. 2). No marked blowflies were recaptured beyond 3 km radius from the release point throughout the 9-day consecutive sampling (Fig. 2).

The recapture rate of released marked blowflies showed a clear decreasing trend with days after release. Of the 43 blowflies recaptured within 6-DAR, 1-DAR recorded the highest recapture rate (32.6%; 14 individuals), followed by 2-DAR (25.6%; 11 individuals), 3-DAR (13.9%; 6 individuals), 4-DAR (11.6%; 5 individuals), 5-DAR (9.3%; 4 individuals) and 6-DAR (7.0%; 3 individuals). The only one blowfly recaptured at 3 km radius from the release point was recaptured at 5-DAR. No blowflies were recaptured after 6-DAR although the trapping lasted for nine days following the initial release (Fig. 2).

In terms of directional movement of the marked blowflies after release, at 1 km radius, the ratio of the 34 recaptured blowflies according to the four cardinal directions (north: east: south: west) was 1 : 2.4 : 1.8 : 1.6. This showed that more blowflies were heading to the east, followed by south and west directions and the least recaptured were in the north direction of the field site. At 2 km radius, out of the eight marked blowflies, there was no fly recaptured in the north, but only one (12.5%) recaptured in the east. Most of the marked blowflies headed to the south (50%; 4 individuals) and southwest (37.5%; 3 individuals). The single blowfly recaptured at 3 km radius from the same release point was also recaptured in the southwest.

## Discussion

This is the first report of blowfly dispersal in a tropical setting, based on mark-release-recapture. The dispersal range of *Chrysomya* blowflies was between 1 to 3 km within 6 days after release. Most of the blowflies (79%) were recaptured at 1 km from the release point throughout the sampling period, whereas approximately 21% were recaptured 2-3 km away from the release point. No blowflies were recaptured at a distance of more than 3 km from the release point. This suggests that *Chrysomya* spp. did not disperse widely, in the range of six days. The daily dispersal distance of < 3 km recorded for *Chrysomya* spp. is similar to the estimated daily dispersal of 2.20 km and 2.35 km reported for *Chrysomyaalbiceps* and *Chrysomyamarginalis*, respectively in the subtropical region of South Africa (Braack and Retief 1986).

The maximum estimated flight distance for blowflies varied depending on species and regions (Braack and Retief 1986). The maximum dispersal distance of tropical blowflies recorded in the present study was 3 km. In a subtropical South Africa study, *Chrysomyaalbiceps* and *Chrysomyamarginalis* were found to disperse up to 37.5 km and 63.5 km, respectively, upon release for a week (Braack and Retief 1986), whereas the maximum dispersal distance of *Chrysomyarufifacies* was 16 km over 12 days in New South Wales, Australia (Gurney and Woodhill 1926). This may be due to each blowfly species having a distinct dispersal rate and flight capability under different climatic conditions (MacLeod and Donnelly 1960, Tsuda et al. 2009).

The recapture rates of blowflies at different distances from the release point were low (0.02-1.1%) throughout the sampling period. This result is similar with the widely-reported low recapture rates in most of the blowfly dispersal studies (see Table 1). Fly dispersal studies using mark-release-recapture are difficult to perform, requiring relatively large number of flies to be released due to low recapture probabilities (Leak 1998). Considering these challenges, our study utilised *Chrysomya* spp. instead of a single species in order to have sufficient numbers for the study. The marked blowflies were not detected beyond 6-DAR, suggesting the longevity of the released blowflies after capativity in the field is less than a week. This, however, may not represent the actual longevity of wild blowflies due to the unknown age of the wild flies used. Hence, this may have partly contributed to the low recapture rate as older flies may fly a shorter distance and die earlier than the younger ones.

The majority of *Chrysomya* spp. blowflies in our study appeared to disperse to the east, followed by south and west at 1 km radius. This could be due to blowflies being attracted towards a small town that is located in the direction of east, where human activities, such as garbaging and farming, are apparent. However, further at 2 km radius from the release point, most of the blowflies were recaptured at the south and southwest direction and the only one marked fly found at 3 km was also in the direction of southwest. The dominant wind direction during the first three days of fieldwork period was northeast, but whether it contributed towards blowfly directional movement remains to be investigated considering the low daily mean wind speed of 0.4-1.0 m/s throughout this first 3 day period (Suppl. material 2). Mixed effects of wind speed on blowfly flight activity have been reported (Mohr 2012). *Calliphoravicina* was capable of initiating voluntary flight at wind speeds below 8.0 m/s, although at above 0.5 m/s, their flight resulted in displacement downwind more commonly than upwind in a wind tunnel (Digby 1958). The log capture rates of *Luciliacuprina* declined linearly at wind speeds above 2.5 m/s (Vogt et al. 1983). This is in contrast with two other studies that showed no significant effect of wind speeds on capture rates of *Chrysomyarufifacies* and *Muscavetustissimu* (Vogt 1986, Vogt 1988).

Detectable mammalian DNA in blowfly guts is only limited to 4 days post-feeding (Lee et al. 2015). Our study suggested that, within this limited period of 4 days, blowflies could possibly sample DNA from the tissues and faeces of mammals and travel up to 1-2 km away from the mammals. This implies that the targeted mammal species, as detected in blowfly-derived DNA, could be present within 1-2 km radius from the site where the blowfly was sampled. This is particularly useful for the monitoring of rare and threatened mammal species, as blowfly-derived DNA can potentially overcome ecological and taxonomical challenges associated with traditional methods (Calvignac‐Spencer et al. 2013, Lee et al. 2016). One advantage of blowfly-derived DNA as compared to other invertebrates could be the short temporal persistence of mammal DNA in blowfly guts (24-96 h) as this precludes mammal species detected in blowfly-derived DNA from being far away from the blowfly sampling location (Lee et al. 2015).

The use of blowfly-derived DNA mammal monitoring tool, together with the knowledge on short temporal persistence of detectable mammal DNA and blowfly dispersal range as indicated from our study, may increase the possibilities of detecting and locating more mammal species in future biodiversity assessment and monitoring. However, there still remains the knowledge gap on blowfly dispersal activities under the influences of surrounding environmental factors, such as solar radiation, rainfall, temperature and wind activity (Von Aesch et al. 2003, Tsuda et al. 2009).

## Conclusions

This study represents the first experimental indication of blowfly dispersal in the Tropics, based on mark-release-recapture method. The estimated location of the targeted mammal via detection from blowfly-derived DNA is likely to be 1-2 km radius and not exceeding 3 km from the location where blowflies were sampled. A more precise estimation of the distance between the targeted mammal and sampled blowflies for monitoring mammals requires more in-depth studies and with inclusion of other environmental factors that could be potentially influencing blowfly dispersal activities and flight range. This certainly warrants future investigation.

## Supplementary Material

C1541305-81E4-56A5-8863-612BC6B04A97 10.3897/BDJ.11.e108438.suppl1 8343839 Supplementary material 1 Supplementary Table 1 Data type table Brief description The number of blowflies recaptured, based on number of days since released and distances of blowflies recaptured from the release point (1-5 km). File: oo_864901.docx https://binary.pensoft.net/file/864901 Ping Shin Lee

0774FA9F-D634-544C-A517-F1C96F5D7658 10.3897/BDJ.11.e108438.suppl2 Supplementary material 2 Supplementary Table 2 Data type table Brief description Records of daily mean wind speed, maximum wind speed and wind direction during the sampling period. File: oo_866731.xlsx https://binary.pensoft.net/file/866731 Ping Shin Lee

## Figures and Tables

**Table 1.** Daily dispersal, dispersal range and recapture rate of adult flies in published mark-release-recapture studies as summarised by dipteran family and species, marking techniques and regions.

Family	Species	Marking techniques	Regions	Daily dispersal	Dispersal range	Recapture rate	References
Calliphoridae	*Calliphoranigribarbis*	Correction fluid	Subtropical (Ikumo-Makka, Japan)	1.250 – 1.789 km	Not estimated	0.014% - 0.029%	Tsuda et al. (2009)
	*Chrysomyaalbiceps*	^32^P-orthophosphate	Subtropical (Kruger National Park, South Africa)	2.20 km	Not estimated	0.1 - 0.45%	Braack and Retief (1986)
	*Chrysomyamarginalis*	^32^P-orthophosphate	Subtropical (Kruger National Park, South Africa)	2.35 km	Not estimated	0.13 - 0.93%	Braack and Retief (1986)
	*Luciliasericata*	Fluorescent dust	Temperate (South West England)	0.11 - 0.15 km	Not estimated	4-14%	Smith and Wall (1998)
	*Phormiaregina*	^32^P-orthophosphate	Subtropical (West Virginia, USA)	Not estimated	9-16 km	< 1%	Schoof and Mail (1953)
	*Callitrogamacellaria*	^32^P-orthophosphate	Subtropical (Savannah, USA)	Not estimated	1.6-4.8 km	0.8-6.0%	Quarterman et al. (1954)
	*Phaenicia* spp.	^32^P-orthophosphate	Subtropical (Savannah, USA)	Not estimated	2.4 km	0-3.8%	Quarterman et al. (1954)
Sarcophagidae	*Sarcophaga* spp.	^32^P-orthophosphate	Subtropical (Savannah, USA)	Not estimated	2.4 km	0-3.3%	Quarterman et al. (1954)
Muscidae	*Muscadomestica*	Fluorescent dust	Tropical (Selangor, Malaysia)	Not estimated	2.05 km	0.016-0.023%	Nazni et al. (2005)
	*Muscadomestica*	^32^P-orthophosphate	Subtropical (Savannah, Georgia)	Not estimated	2.4 km	0.4-3.9%	Quarterman et al. (1954)
	*Muscaautumnalis*	Immunomarking with egg white	Temperate (Prosser, USA)	Not estimated	≤ 0.1 - ≥ 0.45 km	16.3%	Peck et al. (2014)
Drosophilidae	*Drosophila* spp.	Fluorescent dust	Temperate (New Jersey, USA)	Not estimated	0 - > 0.06 km	10%	Worthen (1989)
Tephritidae	*Anastrephaludens*	Fluorescent dye	Tropical (Nuevo Leon, Mexico)	Not estimated	0.1-7 km	0.7-1%	Thomas and Loera-Gallardo (1998)
	*Zeugodacuscucurbitae*	Enamel paint	Subtropical (Ishigaki Island, Japan)	Not estimated	≤ 0.1 km	0.26-8.99%	Hamada (1980)
