# Supplementary material for: Blowfly-derived mammal DNA as mammal diversity assessment tool: Determination of dispersal activity and flight range of tropical blowflies
Source: Biodivers Data J. 2023 Sep 12;11:e108438. doi: 10.3897/BDJ.11.e108438 (PMC10509675; doi:10.3897/BDJ.11.e108438)
Supplement: Supplementary material 1 — Supplementary Table 1 [file bdj-11-e108438-s001.docx]

**Supplementary Table 1** The number of blowflies recaptured based on number of days since released and distances of blowflies recaptured from the release point

(1-5 km).

| Days since release | 1 km | 2 km | 3 km | 4 km | 5 km |
| --- | --- | --- | --- | --- | --- |
| 1 | 14 | 0 | 0 | 0 | 0 |
| 2 | 9 | 2 | 0 | 0 | 0 |
| 3 | 5 | 1 | 0 | 0 | 0 |
| 4 | 2 | 3 | 0 | 0 | 0 |
| 5 | 1 | 2 | 1 | 0 | 0 |
| 6 | 3 | 0 | 0 | 0 | 0 |
| 7 | 0 | 0 | 0 | 0 | 0 |
| 8 | 0 | 0 | 0 | 0 | 0 |
| 9 | 0 | 0 | 0 | 0 | 0 |
